# MicroRNA-720 promotes in vitro cell migration by targeting Rab35 expression in cervical cancer cells

**DOI:** 10.1186/s13578-015-0047-5

**Published:** 2015-09-25

**Authors:** Yunlan Tang, Yi Lin, Chuang Li, Xunwu Hu, Yi Liu, Mingyang He, Jun Luo, Guihong Sun, Tao Wang, Wenxin Li, Mingxiong Guo

**Affiliations:** College of Life Sciences and State Key Laboratory of Virology, Wuhan University, 430072 Wuhan, People’s Republic of China; Department of Pathology, Zhongnan Hospital, Wuhan University, 430071 Wuhan, People’s Republic of China; School of Basic Medical Sciences, Wuhan University, 430071 Wuhan, People’s Republic of China; Department of Respiratory and Critical Care Medicine, Tongji Hospital, Tongji Medical College, Huazhong University of Science and Technology, 430030 Wuhan, China

**Keywords:** miR-720, Cervical cancer cells, Rab35, Cell migration

## Abstract

**Background:**

MicroRNA-720 (miR-720), a nonclassical miRNA, is involved in the initiation and progression of several tumors. In our previous studies, miR-720 was shown to be significantly upregulated in cervical cancer tissues compared with normal cervical tissues. However, the precise biological functions of miR-720, and its molecular mechanisms of action, are still unknown.

**Results:**

Microarray expression profiles, luciferase reporter assays, and western blot assays were used to validate *Rab35* as a target gene of miR-720 in HEK293T and HeLa cells. The regulation of Rab35 expression by miR-720 was assessed using qRT-PCR and western blot assays, and the effects of exogenous miR-720 and Rab35 on cell migration were evaluated in vitro using Transwell^®^ assay, wound healing assay, and real-time analyses in HeLa cells. The influences of exogenous miR-720 on cell proliferation were evaluated in vitro by the MTT assay in HeLa cells. In addition, expression of E-cadherin and vimentin associated with epithelial-mesenchymal transition were also assessed using western blot analyses after transfection of miR-720 mimics and Rab35 expression vectors. The results showed that the small GTPase, Rab35, is a direct functional target of miR-720 in cervical cancer HeLa cells. By targeting Rab35, overexpression of miR-720 resulted in a decrease in E-cadherin expression and an increase in vimentin expression and finally led to promotion of HeLa cell migration. Furthermore, reintroduction of Rab35 3′-UTR(−) markedly reversed the induction of cell migration in miR-720-expressing HeLa cells.

**Conclusions:**

The miR-720 promotes cell migration of HeLa cells by downregulating Rab35. The results show that miR-720 is a novel cell migration-associated gene in cervical cancer cells.

**Electronic supplementary material:**

The online version of this article (doi:10.1186/s13578-015-0047-5) contains supplementary material, which is available to authorized users.

## Background

Discovered in 1993, microRNAs (miRNAs) are a class of short, non-coding RNAs that are highly efficient gene expression regulators in various cellular processes [1–5]. They modulate gene expression predominantly through an interaction with the 3′-UTR of their target mRNAs [6, 7]. Increasing evidence suggests that dysfunctions of miRNAs are involved in the initiation and progression of cancer [8, 9], as well as in animal developmental processes [10]. It has been reported that miR-720 is not a classic miRNA, but is probably a fragment of tRNA [11]. Hara et al. identified miR-720 as a novel miRNA regulator in the differentiation of dental pulp cells [12]. Other studies suggested that circulating miR-720 was a novel serum biomarker in some tumors, such as colorectal cancers [13] and myelomas [14]. Furthermore, miR-720 could act as a colorectal cancer-promoting factor, and could be a marker for the prognosis of colorectal cancer [15]. Previous results have reported that miR-720 is also frequently decreased in breast cancer and functions as an anti-metastatic gene by downregulating TWIST1 [16]. Our previous studies suggested that miR-720 expression is significantly upregulated in cervix uteri squamous cell carcinoma tissues, when compared with normal cervix uteri tissues [17]. Cervical cancer is the third most commonly diagnosed cancer, and the fourth most common type of cancer among women, worldwide [18]. However, the roles of miR-720 in the initiation and progression of cervical cancer are still largely unknown.

The small GTPase, Rab35, is a member of the RAS oncogene family. It regulates many essential cellular processes, such as recycling from endosomes for neurite outgrowth [19–25], exosome release [26], cytokinesis [27], and actin cytoskeleton organization [28–31] by cycling between a GTP-bound active form and a GDP-bound inactive form [32]. In addition, Rab35 is involved in the early stage of FcγR-mediated phagocytosis in macrophages [33] and also acts as a regulator of vesicle transport required specifically for phagocytosis [34]. Although Rab35 has been shown to regulate various cellular processes, its precise roles in these processes are not fully understood. Moreover, several recent studies have reported that dysfunctions of Rab35 also exert vital functions, such as regulation of myoblast fusion and cadherin-dependent adherens junction formation [35]. Some studies showed that Wnt5a promotes breast cancer cell migration via the Dvl2/Rab35/Rac1 signaling pathway [36], and Rab35 maintains cadherins at the cell surface to promote cell–cell adhesion [37].

In the present study, we therefore sought to determine the role of miR-720 in cervical cancer cells. We first defined miR-720 as a new, in vitro cell migration-associated miRNA in HeLa cells. And overexpression or inhibition of miR-720 did not significantly affect HeLa cell proliferation. Moreover, we identified Rab35, a key regulator of endosomal membrane trafficking, as a direct and functional target of miR-720. The newly identified miR-720/Rab35 axis provides a molecular mechanism for abnormal cell migration in cervical cancer cells via its effects on the expression of an epithelial marker, E-cadherin, and a mesenchymal marker, vimentin.

## Results

### miR-720 promotes cell migration but does not affect cell proliferation in HeLa cells

**Fig. 1:**
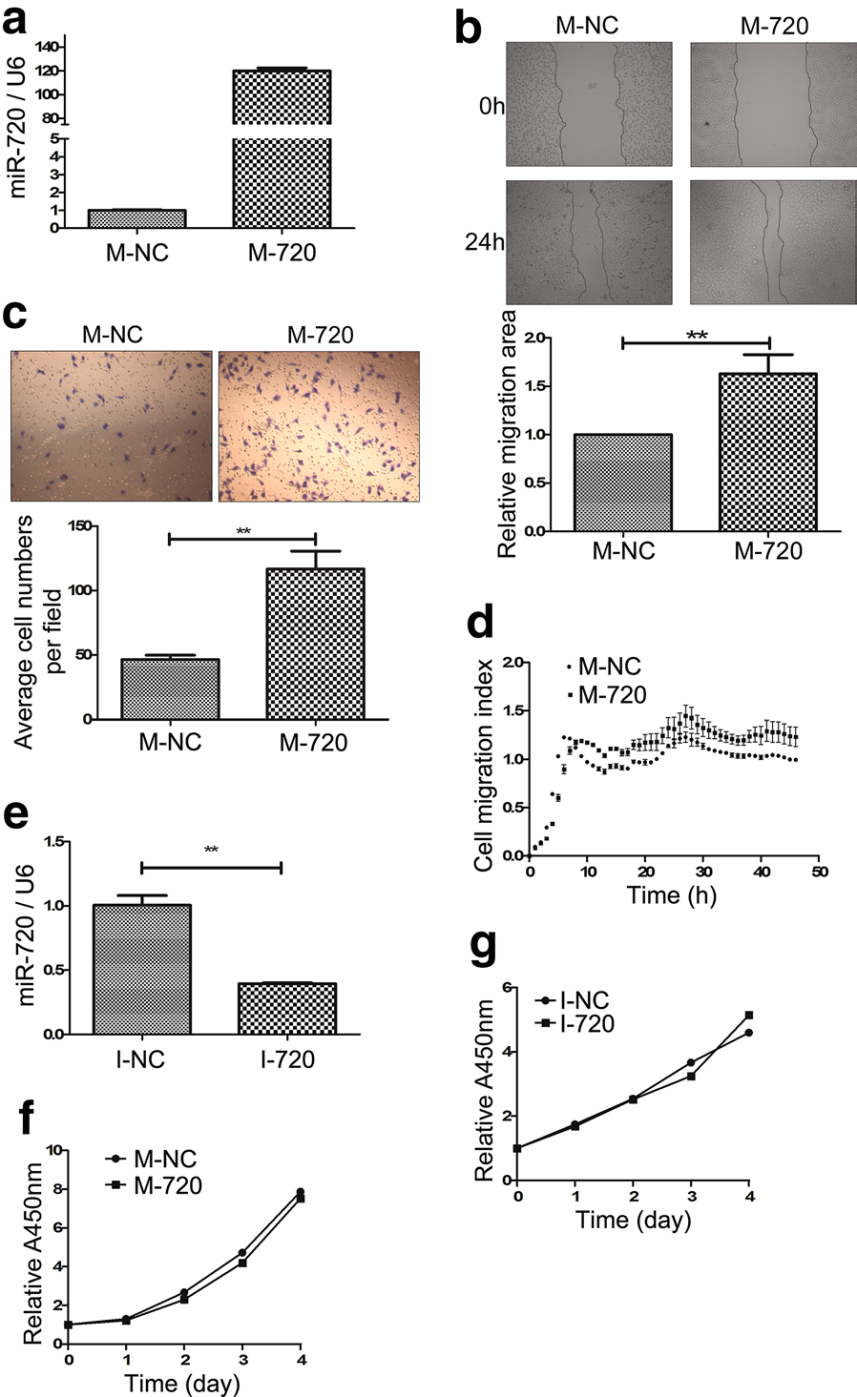
MiR-720 promotes cell migration but does not affect cell proliferation in HeLa cells. **a** The qRT-PCR analysis of miR-720 expression indicated that HeLa cells transfected with miR-720 mimics (M-720) showed a significant increase in miR-720 expression compared with cells transfected with the microRNA mimic negative control (M-NC). **b** Representative photographs of wound healing assays (100×) show that HeLa cells transfected with miR-720 mimics result in a significant improvement in wound healing ability. The statistical results of cell migration area measured by Image J software demonstrate a significant difference in cell migration ability caused by overexpression of miR-720. **p *<* 0.01. **c** Transwell^®^ migration assay shows that the upregulation of miR-720 dramatically enhances cell migration ability. The *top panel* shows representative photographs of the Transwell^®^ migration assay and the *bottom panel* shows the statistical results. **p *<* 0.01. **d** Twenty-four hours after transfection, real-time cell analysis (RTCA) experiments were performed to contrast cell migration indices between cells transfected with miR-720 mimics and the microRNA mimic negative control. The results show that after 8 h, the migration indices of cells transfected with miR-720 mimics were greater than the controls. **e** The results of qRT-PCR analysis of miR-720 expression show that the miR-720 inhibitors can effectively knockdown miR-720 in HeLa cells. **p *<* 0.01. The (3-4,5-dimethyl-2-thiazolyl)-2,5-diphenyl-2-H-tetrazolium bromide (MTT) assays show upregulation (**f**) or downregulation (**g**) of miR-720, does not show an effect on cell proliferation viability in HeLa cells

In our previous study, we found the expression level of miR-720 was significantly upregulated in cervical cancer tissues compared to normal adjacent tissues [17]. To further explore its functions in the development and progression of cervical cancer, we assessed the influence of miR-720 on cell migration and cell proliferation in HeLa cells, by gain- and loss-of-function analyses. First, we showed that miR-720 mimics could lead to a significant increase in miR-720 expression (Fig. 1a). Then, cell migration assays were performed after HeLa cells were transfected for 24 h with the miR-720 mimic (M-720) or the miR-mimic negative control (M-NC). Overexpression of miR-720 in HeLa cells significantly promoted migration ability, as determined by wound healing assays, showing that the abilities of migrated cells filling a scratch was significantly enhanced in miR-720-overexpressed cells than the M-NC cells (*p* < 0.01) (Fig. 1b). The induction effect of miR-720 on cell migration was further confirmed by Transwell^®^ assays. The representative photographs of Transwell^®^ migration, plus the histogram of the results, both showed that the M-720 had a significant improvement on cell migration (Fig. 1c). A real-time cell analysis is a new method to accurately detect cell migration in real time. Using this method, we further demonstrated that upregulation of miR-720 could induce cell migration in HeLa cells (Fig. 1d).

In addition, inhibition by miR-720 inhibitors resulted in a dramatic decrease in miR-720 expression (Fig. 1e). We therefore used these transfected cells to conduct 3-4,5-dimethyl-2-thiazolyl)-2,5-diphenyl-2-H-tetrazolium bromide (MTT) assays to assess the effects of miR-720 on cell proliferation. The results showed that neither the miR-720 mimic nor the miR-720 inhibitor had significant effects on cell proliferation (Fig. 1f, g). Thus, we next studied the role of miR-720 in cell migration of HeLa cells.

### Identification of the miR-720 targets by integrative bioinformatics analysis

**Fig. 2:**
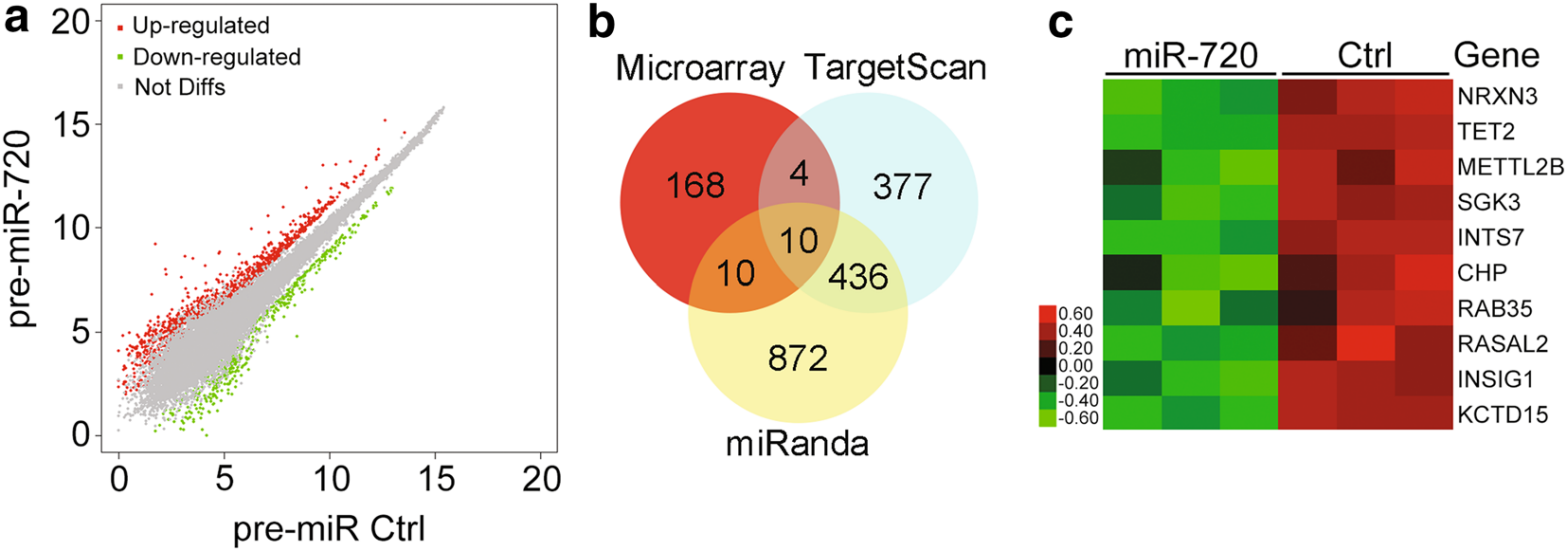
Identification of miR-720 targets. **a** Microarray assays were performed on HEK293T cells transfected with pre-miR-720 and the pre-miR-control. The *red dots* represent genes upregulated with a ≥2-fold change, the *green dots* represent genes downregulated with a >0.5-fold change, and the *gray dots* indicate genes with expression levels ranging from −0.5-fold change to +2-fold change in the scatterplot. **b** The *Venn diagram* shows the relationships between the mRNA microarray and two miRNA-target prediction algorithms on the quantity of miR-720 targets. The TargetScan program and the miRanda program predicted that 827 candidate genes and 1328 candidate genes, respectively, were possible targets of miR-720. Expressions of 192 genes in HEK293T cells were changed ≥2-fold with a p value cut-off of 0.05 by ectopic expression of pre-miR-720. Among these 192 genes, 14 and 20 were predicted by TargetScan 5.1 and miRanda, respectively. Among these 20 genes, 10 were classified as the intersection targets. **c** The heat map shows the change in expression levels of 10 genes with overexpression of miR-720

In order to characterize the molecular mechanism of miR-720 on cell migration, the potential mRNA targets of miR-720 were identified. To investigate the potential target genes and their binding sites on the seed region of miR-720, we used the TargetScan program (http://www.targetscan.org/) [7, 38, 39] and the miRanda program (http://www.microrna.org/) [40]. The TargetScan and miRanda program predicted respectively 827 and 1328 candidate genes, which were possible targets of miR-720 (Fig. 2b).

Recent studies have shown that miRNAs can reduce the levels of many of their target transcripts, and not just protein expression deriving from these transcripts [41]. Based on these observations, we used a high throughout genome mRNA microarray to identify potential target genes of miR-720. We performed global microarray gene expression profiling using the Human Genome U133 Plus 2.0 Array (Affymetrix, Santa Clara, CA, USA) in HEK293T cells transfected with pre-miR-720 or negative control mimics. Twenty-four hours after transfection, the expression level of miR-720 (relative to endogenous U6 RNA) in HEK293T cells was determined by qRT-PCR. The expression level of miR-720 was increased about 550-fold as compared to the negative control. The microarray results showed that when compared with the controls, 216 probes, representing 195 genes (three of these genes are still unnamed and not included) were downregulated by ≥2-fold (*p* < 0.05) in pre-miR-720 transfected HEK293T cells (Fig. 2a; Additional file 1: Table S1). Using a Gene List Venn Diagram, we identified 10 potential target genes as *CHP, INSIG1, INTS7, KCTD15, METTL2B, NRXN3, PER2, RAB35, SGK3,* and *TET2* among the microarray results and the putative miR-720 target gene list (as predicted by TargetScan and miRanda) (Fig. 2c).

### Identification of miR-720 targets by the luciferase reporter assay

**Fig. 3:**
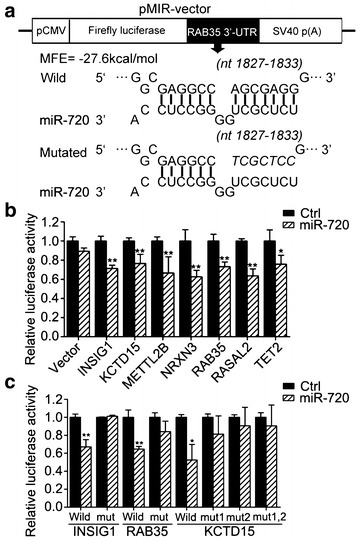
Dual luciferase reporter assays verify that miR-720 regulates its targets by binding directly to their 3′-UTR. **a** The diagram shows that 3′-UTR of Rab35 was cloned downstream into the open reading frame of the pcDNA3.0-Vector, and also displays the detailed binding sites or the mutated binding sites of the 3′-UTR for miR-720 targeting; **b** The results of analysis indicate that the relative luciferase activities of the pMIR-REPORT vector containing wide-type 3′-UTR of *INSIG1, KCTD15, METTL2B, NRXN3, Rab35, RASAL2,* or *TET2* could be decreased by upregulating miR-720; Firefly luciferase activity was normalized to Renilla luciferase activity. The relative luciferase expression level is expressed as the mean ± standard deviation (SD). Three independent experiments were performed, and representative data are shown. *p < 0.05 shows the significant difference. **c** Normalized luciferase activity in cells transfected with reporter vector containing mutant type 3′-UTR of *KCTD15*, *INSIG1*, or *RAB35* could not be affected by overexpression of miR-720 compared with cells transfected with wild type 3′-UTR. The relative luciferase expression level is expressed as the mean ± standard deviation (SD). Three independent experiments were performed, and representative data are shown. *p < 0.05 shows the significant difference

Using luciferase reporter assays, we next sought to verify direct regulation of these candidate targets by miR-720. Among these candidate target genes, except for *KCTD15* with two predicted miR-720 binding sites in 3′-UTR, the rest of the target genes had only a predicted target site in 3′-UTR. We subcloned the partial 3′-UTRs containing the miR-720-binding sites of these candidate target genes, such as Rab35, into the luciferase-based reporter vector pMIR-REPORT (Ambion, Austin, Texas, US), and cotransfected the reporter constructs in HEK293T cells with the pre-miR-720 precursor or negative control (Fig. 3a). Among these reporter constructs, miR-720 significantly suppressed the luciferase activity of the reporter vector containing binding sites in *INSIG1, KCTD15, METTL2B, NRXN3, Rab35, RASAL2,* or *TET2*, but not in *CHP, PER2,* or *SGK3* for miR-720 targeting (Fig. 3b). To further confirm the predicted miRNA binding sites, seven base pair mutations of the miR-720 binding sites on the *INSIG1, KCTD15, METTL2B, NRXN3, Rab35, RASAL2,* and *TET2* genes were generated by site-directed mutagenesis. Because *KCTD15* has two predicted binding sites in its 3′-UTR, we performed molecular cloning for single site mutations to obtain vectors named MUT1-KCTD15, MUT2-KCTD15, and double site mutation vectors named MUT1,2-KCTD15. As expected, once the miR-720 binding sites in the 3′-UTR of these candidate target mRNAs were mutated, the luciferase activities could not be suppressed by miR-720 (Fig. 3c). Taken together, the results of these reporter assays showed that miR-720 could directly bind to the 3′-UTR of *INSIG1*, *KCTD15*, and *RAB35*.

### Rab35 is a target of miR-720 in HeLa cells

**Fig. 4:**
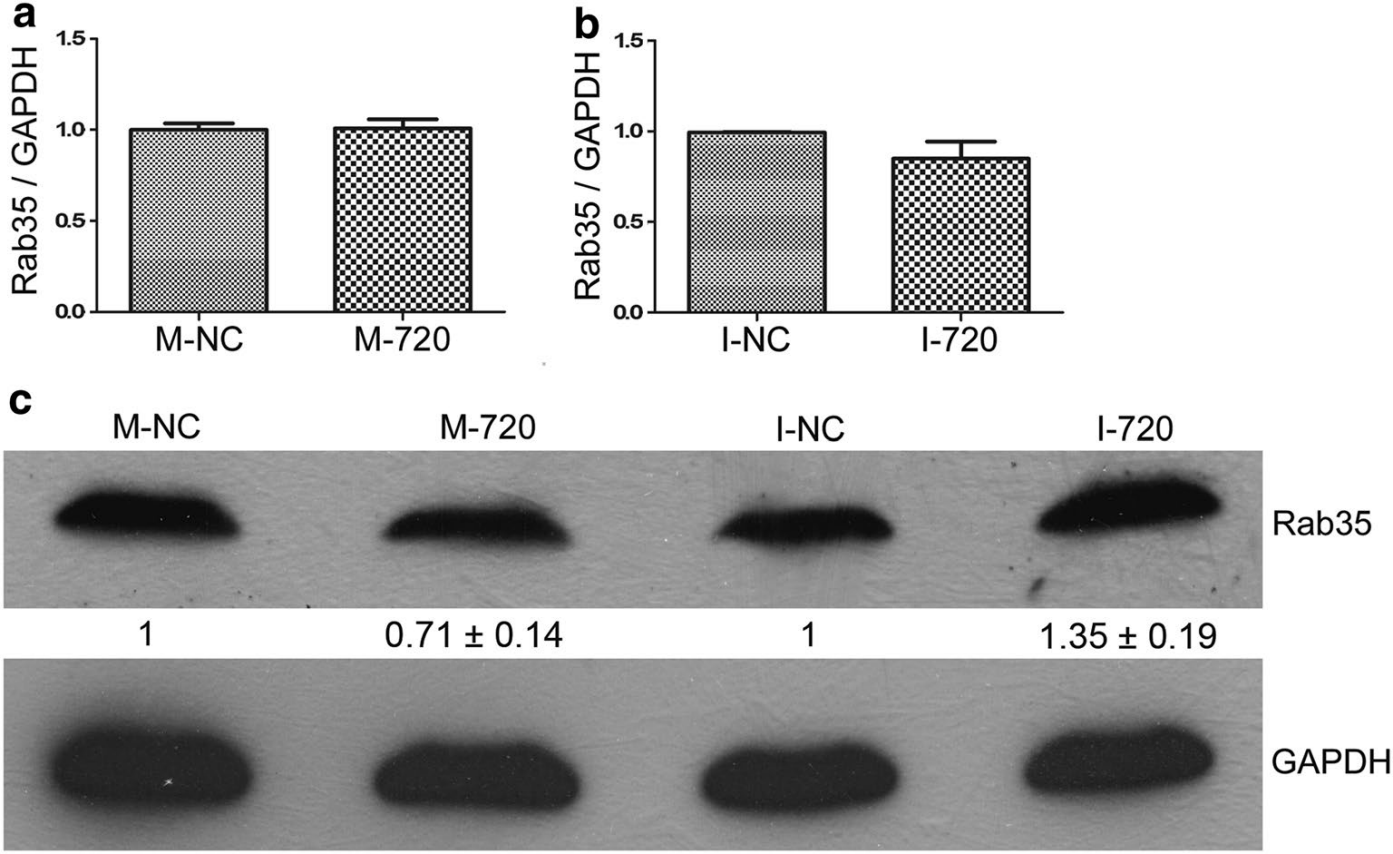
Rab35 is negatively regulated by miR-720 at the translational level in HeLa cells. Transfection of miR-720 mimics (**a**) or miR-720 inhibitors (**b**) in HeLa cells did not significantly affect Rab35 mRNA expression levels, as assessed by qRT-PCR analysis. **c** Representative photographs of Rab35 protein expression detected by western blotting, and the results of gray intensity analysis using Image J software showed that Rab35 protein levels were negatively regulated by miR-720

The studies suggested that *INSIG1* is an insulin-induced gene and encodes an endoplasmic reticulum (ER) membrane protein that plays a critical role in regulating cholesterol concentrations in cells [42–46]; and Kctd15 has a role in regulating the neural crest formation in the embryo [47, 48]. And Rab35 was involved in breast cancer cell migration processes [36] and promotes cell–cell adhesion [37]. Because miR-720 could induce cell migration in HeLa cells, we focus our initial research emphasis on Rab35. Firstly we further confirmed whether the mRNA and protein expression levels of *Rab35* could have been regulated by miR-720 in cervical cancer HeLa cells. The results showed that mRNA expression of Rab35 was not significantly affected by both the overexpression and suppressed expression of miR-720 (Fig. 4a, b). However, the protein level of Rab35 was negatively regulated by miR-720 in HeLa cells as expected (Fig. 4c). Thus, we propose that miR-720 plays a role in the regulation of Rab35 at the translational level in HeLa cells.

### Rab35 can suppress cell migration of HeLa cells

**Fig. 5:**
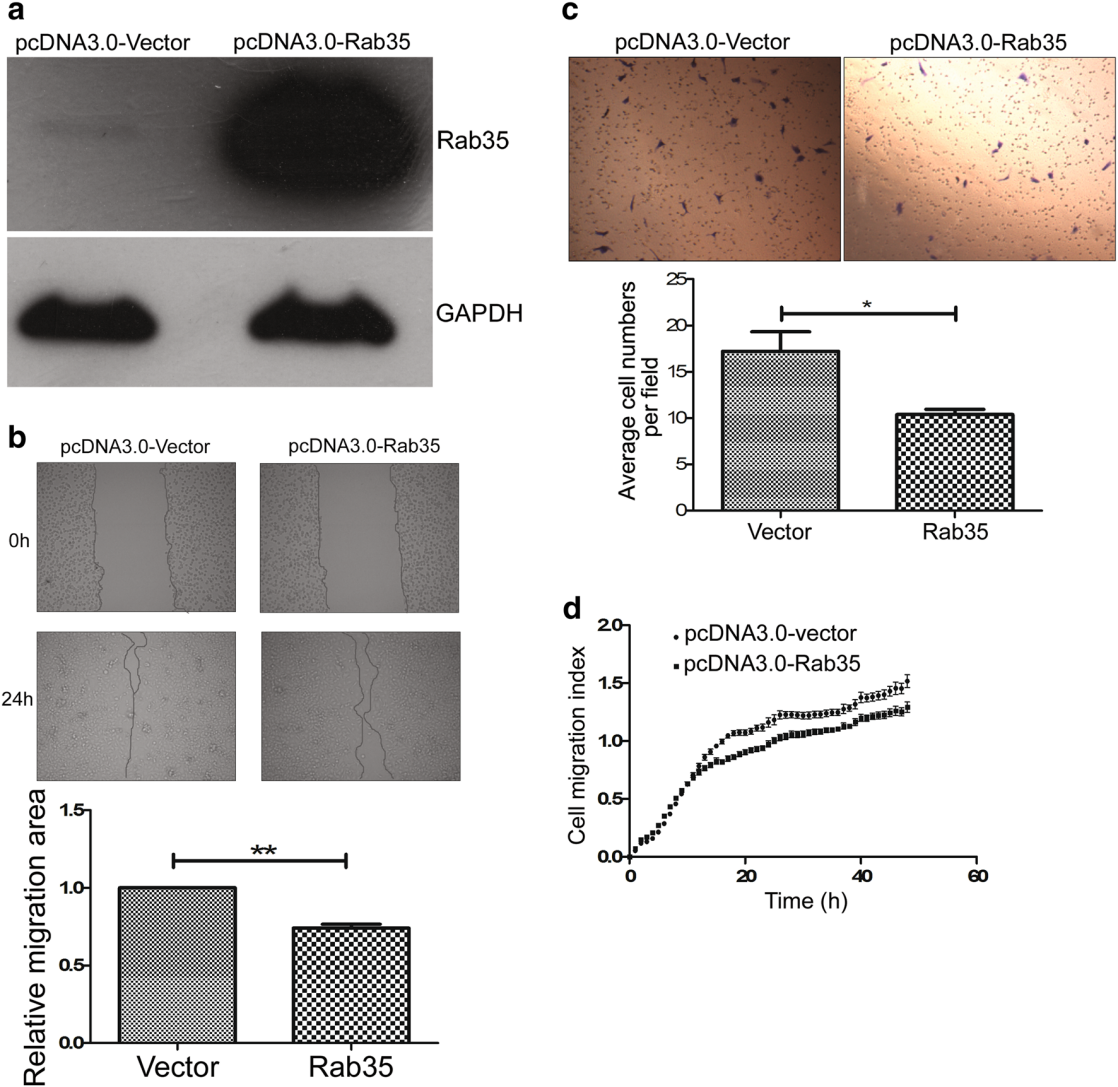
Rab35 has the opposite effect on the migration of HeLa cells, relative to miR-720. **a** Representative western blot assays show that transfection with the constructed vector pcDNA3.0-Rab35 results in a high expression level of Rab35. **b** Representative photographs of wound healing assays show that the forced expression of Rab35 decreased cell wound healing ability. The statistical results of cell migration area measured by Image J software demonstrate a significant reduction in cell migration caused by overexpression of Rab35. **p *<* 0.01. **c** Representative photographs of Transwell^®^ migration assays and the statistical results show that the forced expression of Rab35 led to a dramatic decrease in cell migration. *p *<* 0.05. **d** Real-time cell analysis shows that overexpression of Rab35 decreased the cell migration index when compared with the corresponding negative control

Then we wondered whether Rab35 had negative effects on cell migration in HeLa cells compared with miR-720. So we constructed a miR-720 insensitive Rab35 expression vector (pcDNA3.0-Rab35) that only included the coding sequence of Rab35 on the base of the pcDNA3.0-Vector, to escape miR-720 targeting. The overexpression level of this vector was assessed by western blotting, and the results showed that the vector successfully induced Rab35 overexpression (Fig. 5a). Then, we assayed the impact of forced expression of Rab35 on cell migration of HeLa cells. Using the wound healing assay, the overexpression of Rab35 inhibited the healing of the scratch when compared to the control group transfected with the empty pcDNA3.0-Vector (Fig. 5b). In the other two types of cell migration assays (Transwell^®^ migration assay and real-time cell analysis), we consistently found that Rab35 had the ability to inhibit cell migration of HeLa cells (Fig. 5c, d). These results showed that Rab35 has an opposite effect on HeLa cell migration, in comparison with miR-720, indicating that it may be a functional target of miR-720 in HeLa cells.

### miR-720 mediates cell migration by targeting Rab35 in HeLa cells

**Fig. 6:**
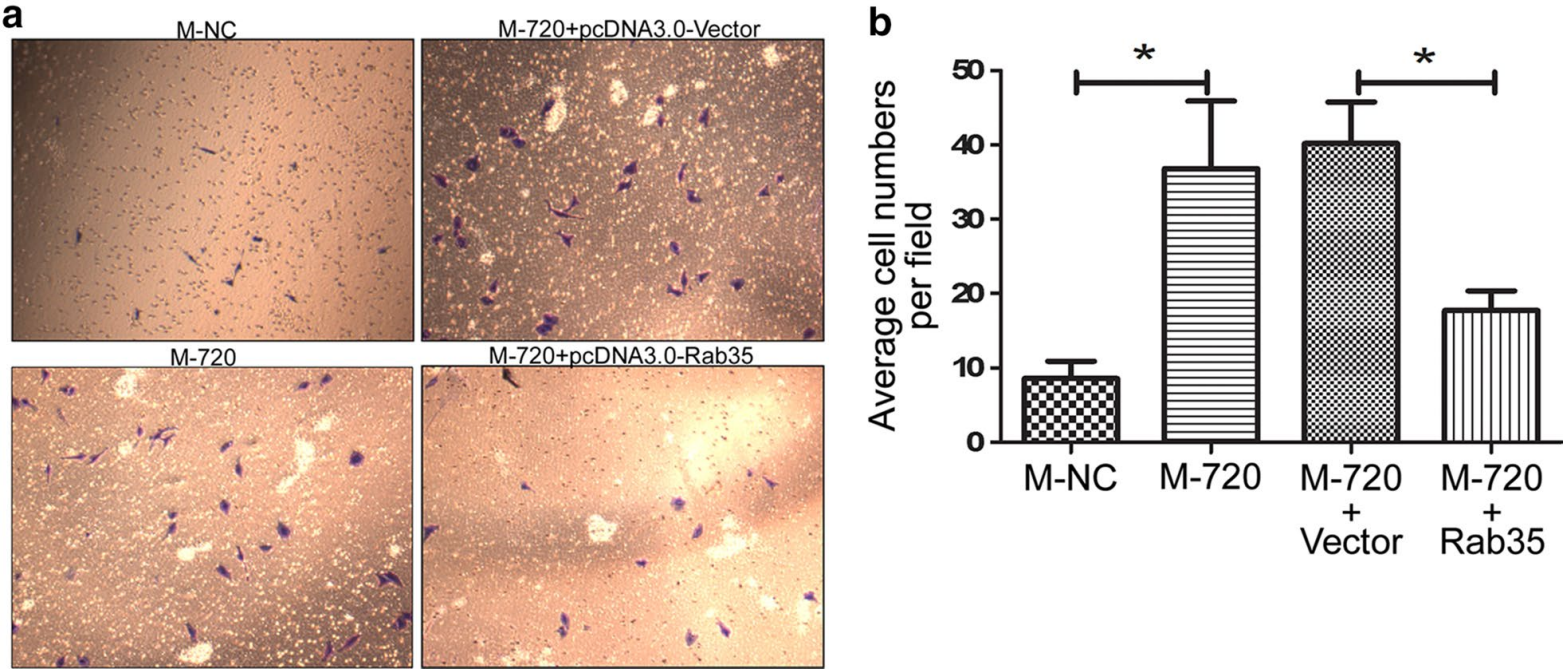
Restoration of Rab35 expression reverses the promoting effect of miR-720 on cell migration. Representative photographs (**a**) and statistical results (**b**) of Transwell^®^ assays show that while transfecting miR-720 mimics could lead to a significant increase in cell migration ability compared with cells transfected with the microRNA mimic negative control, cotransfecting pcDNA3.0-Rab35 and miR-720 mimics resulted in a dramatic decrease in cell migration ability compared with the negative group cotransfected with miR-720 mimics and the pcDNA3.0-Vector. *p *<* 0.05

In the previous experiments, overexpression of miR-720 could promote cell migration of HeLa, and forced expression of Rab35 could suppress cell migration. To further demonstrate whether Rab35 is the actual functional target of miR-720 acting on cell migration of HeLa cells, we cotransfected the miR-720 mimic and the miR-720 insensitive Rab35 vector into HeLa cells, then conducted a Transwell^®^ migration assay. Even though there is overexpression of miR-720, the miR-720 insensitive Rab35 vector can still restore Rab35 expression and antagonize the positive effect of miR-720 on cell migration of HeLa cells, finally resulting in inhibition of cell migration, compared with the negative control group transfected with the miR-720 mimic and the empty pcDNA3.0-Vector (Fig. 6a, b). Thus, we postulate that miR-720 exerts its positive function on cell migration of HeLa cells by negatively regulating Rab35, which can suppress cell migration of HeLa cells.

### Molecular mechanism of the miR-720 effect on cell migration of HeLa cells

**Fig. 7:**
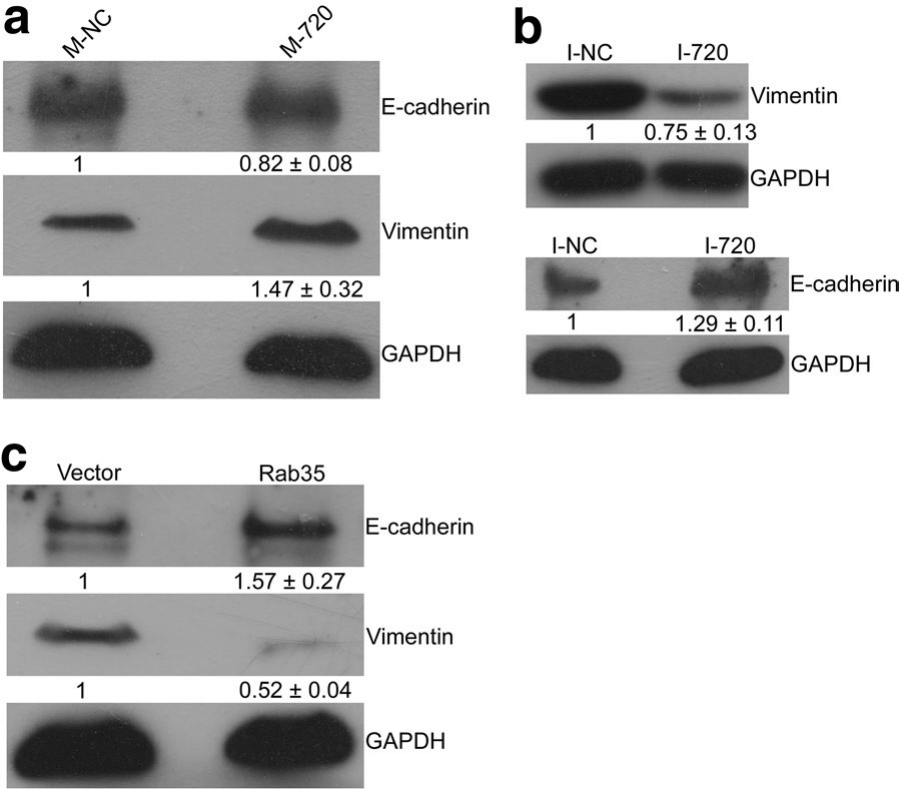
Detection of E-cadherin and vimentin expression by western blotting. **a** HeLa cells transfected with miR-720 mimics show a significant increase in vimentin protein expression and a dramatic decrease in E-cadherin protein expression. Transfection of miR-720 inhibitors (**b**) or pcDNA3.0-Rab35 (**c**) had a negative effect (E-cadherin was increased and vimentin was decreased), in contrast to transfection of miR-720 mimics in HeLa cells

We next sought to characterize the mechanism of action involved in miR-720 induction of HeLa cell migration. Epithelial-to-mesenchymal transition (EMT) is an important process in morphogenesis and tissue repair, and this process is often abnormally activated in cancer cells, which will promote their malignant and stem cell characteristics and finally accelerate cell migration [49]. To determine if EMT was aberrantly changed, we assessed the alterations in E-cadherin protein expression, which is a well-established marker for epithelial cells, and vimentin, which is a marker of mesenchymal cells, in the transfected HeLa cells used in the previous functional study [50]. The results showed that the forced expression of miR-720 upregulated vimentin and downregulated E-cadherin (Fig. 7a). Correspondingly, miR-720 inhibition and overexpression of Rab35 had the opposite effects on E-cadherin and vimentin levels; E-cadherin was increased and vimentin was decreased (Fig. 7b, c). Collectively, these results suggest that the intrinsic mechanism of miR-720 promotion on cell migration by downregulation of Rab35 is essentially through the activation of the EMT in HeLa cells.

## Discussion

Tumor metastasis remains a major cause of cancer-related death. Metastasis is comprised of various sequential steps, involving cancer cell dissemination, cancer cell spreading, and formation of secondary tumors in other organs and tissues [51, 52]. The first essential step of tumor metastasis and invasion is the change in the expression of cell adhesion molecules (CAM), and the breakdown and remodeling of the extracellular matrix (ECM), resulting in the ability of cancer cells to metastasize. Increasing evidence suggests that miRNAs play critical roles in the regulation of cell migration and invasion, as coordinated regulators of ECM remodeling during cancer cell migration and invasion [53]. We previously reported that miR-720 expression is significantly upregulated in cervix uteri squamous cell carcinoma tissue when compared to normal cervix uteri tissue, indicating that miR-720 is closely related to the pathological processes of cervical cancer [17]. Some studies have reported that miR-720 impedes cell invasion and migration, and then inhibits metastasis in breast cancer by directly targeting TWIST1 [16]. In the present study, we showed that overexpression of miR-720 leads to a reduction of E-cadherin and an increase of vimentin protein levels in cervical cancer cell lines, which indicates that miR-720 can induce EMT in cervical cancer cells and promote cell migration. In combination with our previous studies and reports from other investigators, we propose that miR-720 may be an important regulator of cell migration and invasion in various cancers, in spite of its functional differences that are dependent on distinct cellular microenvironments. It was reported that individual miRNA has the potential to regulate hundreds of targets [54]. In addition, a miRNA can not only directly affect the levels of target mRNA but also indirectly affect the levels of target mRNA, in other words, a miRNA can have secondary effects or even tertiary effects on its target mRNA [55]. Besides, the effects of a miRNA on regulating its targets crucially depends on its expression levels and the regulation happened to have a cell-type dependent effect [56–58]. Therefore, we propose that miR-720 may regulate the cell migration processes in various cancer cells by distinct molecular mechanism.

Since miR-720 was discovered, it has been widely studied as a miRNA. A study showed that miR-720 could negatively regulate p63 involved in an autoregulatory feedback loop, which then contributed to epithelial development processes [59]. The miR-720 could suppress cell migration and invasion in breast cancer by directly targeting TWIST1 [16]. Furthermore, miR-720 participates in the control of the stem cell phenotype of human dental pulp cells by directly repressing NANOG levels [12]. miR-720 is closely related to the pathological processes of various tumors [17]. A more recent study showed that overexpression of miR-720 in primary human CD8+ T cells inhibits the T cell receptor (TCR) stimulation-induced proliferation in patients with chronic hepatitis B virus (HBV) infection, which suggests that therapies targeting miR-720 may help restore impaired immunity in chronic HBV-infected patients [60]. However, another study reported that miR-720 is probably a fragment from a tRNA, rather than an miRNA [11]. And other report showed that, as a tRNA-derived miRNA, miR-720 is one of the four most abundant MCF7 extracellular vesicle miRNAs, suggesting that miR-720 may be a new biomarker in breast cancer cells [61]. Although there is controversy over whether miR-720 is a miRNA or a tRNA-derived RNA fragment (tRF), functional studies of miR-720 will provide valuable information concerning the possible clinical significance of this small non-RNA molecule.

As a key regulator of endosomal membrane trafficking and the actin cytoskeleton, Rab35 in this study was found to be directly regulated by miR-720 at the posttranscriptional level. The small GTPase, Rab35, a member of the RAS oncogene family, regulates many essential cellular processes, such as recycling from endosomes, exosome release, cytokinesis, and actin cytoskeleton organization by cycling between a GTP-bound active form and a GDP-bound inactive form. Active Rab35 controls recycling pathways to increase cell adhesion and decrease cell migration [32]. It is noteworthy that many studies have demonstrated a close relationship between Rab35 and Arf6, which is also a small GTPase. Both these two small GTPases are involved in functionally related cell processes, such as cell migration and cytokinesis, endocytosis, actin remodeling, sorting, and recycling of adhesion molecules. However, the activities of Rab35 and Arf6 are inversely coordinated [24, 27, 62, 63]. To the best of our knowledge, this is the first study to provide experimental evidences that Rab35 is posttranscriptionally regulated by miR-720, which then contributes to the regulation of cell migration. Based upon these observations, it is important to further validate the functions of the miR-720/Rab35 axis in tumor metastasis and invasion in animal models and clinical tissues in future studies.

## Conclusions

Our results show that miR-720 promotes cell migration in cervical cancer cells by directly targeting Rab35. Targeting the miR-720/Rab35 axis is therefore a promising therapeutic strategy for cervical cancer treatment.

## Methods

### Cell culture

HeLa cells and HEK293T cells were obtained from the Type Culture Collection of the Chinese Academy of Sciences (Shanghai, China). HeLa cells were cultured in Minimum Essential Medium (Gibco/Life Technologies, US) and HEK293T cells were incubated in Dulbecco’s Modified Eagle’s Medium (Gibco/Life Technologies, US). The base medium was supplemented with 10 % fetal bovine serum (Gibco/Life Technologies, US) to make the complete growth medium, and the cells were incubated at 37 °C with 5 % CO_2_.

### Oligonucleotide transfection

The pre-miR-720, miR-720 mimics, inhibitor, and corresponding controls were designed and synthesized by RiboBio (Guangzhou, China). HEK293T cells were transfected with pre-miR-720 or pre-miR-control at a final concentration of 100 nM. HeLa cells were transfected with mimics at a final concentration of 100 nM, or transfected with inhibitor at a final concentration of 200 nM using Lipofectamine^®^ RNAiMAX reagent (Invitrogen, US), following the manufacturer’s instructions. After 48 h, the cells were harvested for further analysis.

### RNA extraction and quantitative real-time PCR

Total RNA was extracted using TRIzol^®^ Reagent (Life Technologies, US) according to the manufacturer’s protocol. One µg of total RNA was reverse transcribed into cDNA with a FastQuant RT Kit (Tiangen Biotech, Beijing, China). Quantitative analyses of miR-720 were performed using the Bulge-LoopTM miRNA quantitative RT-PCR primer (RiboBio, Guangzhou, China) and/or a S-Poly(T) real-time PCR assay of microRNAs assays [64], and SYBR Select Master Mix (Life Technologies) on an ABI Prism 7500 real-time PCR system (Applied Biosystems, Foster City, CA, USA). Quantitative analysis of Rab35 was performed using designed qPCR primers (forward, 5′-ACGACCACCTCTTCAAGCTG-3′; reverse, 5′-CCGGATCTTGAAATCCACTC-3′) and the same SYBR Select Master Mix with miR-720 qPCR. PCR was performed using 20 µL reaction mixture in triplicate at 95 °C, for 10 min, followed by 40 cycles of 95 °C for 10 s, 60 °C for 34 s, and 72 °C for 30 s. U6 snRNA was used as an internal control for miRNA analysis, and glyceraldehyde 3-phosphate dehydrogenase (GAPDH) mRNA was used as an internal control for quantitative analysis of mRNA. The results were calculated using the 2^−ΔΔCT^ method.

### Microarray analysis

After the experimental HEK293T group was transfected with pre-miR-720 and the control HEK293T group was transfected with the pre-miR-control for 24 h, the HEK293T cells were lysed with TRIzol^®^ reagent (Life Technologies, US) and total RNA was extracted according to the manufacturer’s protocol. The RNA samples were then sent to the Shanghai Biotechnology Corporation to perform microarray analysis (Affymetrix GeneChip^®^ Human Genome U133 Plus 2.0 Array). Each group sample set was comprised of three replicates. The Affymetrix GeneChip^®^ system was used for hybridization, staining, scanning, and imaging of the arrays. Raw data were analyzed with the Affymetrix GeneChip^®^ Operating Software (GCOS1.4) using the manufacturer’s default analysis settings and global scaling as the normalization method. One-way analysis of variance (ANOVA) was performed with GeneSpring 7.31 software (Agilent, Santa Clara, CA, USA) to identify genes whose expression changed significantly between the pre-miR-720 group and the control.

### Luciferase reporter assay

The putative binding sites of the 3′-UTR of the human genes for miR-720 targeting were predicted by TargetScan Human (http://www.targetscan.org/) computational methods.

The partial 3′-UTR fragment (about 250 bp) of these potential targets, including predicted binding sites, as our previous report [65], were separately cloned into the pMIR-REPORT™ miRNA Expression Reporter Vector (Applied Biosystems) at the *Mlu* I/*Hin*d III site. The primers were designed as Table 1.

**Table 1:** Primers used in this study

Name	Sequences (5′–3′)
3′UTR-CHP-S	CAAAACGCGTCTGTCAACACAAACCTGC
3′UTR-CHP-A	CGTAAAGCTTGCCAGACCCCTGAACTAT
3′UTR-INSIG1-S	GCTTACGCGTGAGGGAAATGTCTTGGAG
3′UTR-INSIG1-A	CGCCAAGCTTGATGTTCAAATCTGGTAG
3′UTR-INTS7-S	ATCGACGCGTACAGTTTGGTTTTTCATA
3′UTR-INTS7-A	ACTCAAGCTTAAACAAGAAAGAACAATC
3′UTR-KCTD15-S	CAACACGCGTATCGTCACAGGAGAAGGT
3′UTR-KCTD15-A	AGACAAGCTTTCCAACCCCGCCCCAAC
3′UTR-METTL2B-S	ATATACGCGTCCCGTTGTGTTTCCGAGC
3′UTR-METTL2B-A	CGGCAAGCTTAGCCCTTCACTAACCCCT
3′UTR-NRXN3-S	ACTTACGCGTCTGAGGGGAAAAATGGCT
3′UTR-NRXN3-A	TAACAAGCTTTGGCTGGGTGAATGGAC
3′UTR-RAB35-S	TTAAACGCGTAAGGAGGGTGCTGTGGAG
3′UTR-RAB35-A	ACACAAGCTTCCCATAACCCCCATTCAT
3′UTR-SGK3-S	ATATACGCGTGAGACCATCCTGGGCAAC
3′UTR-SGK3-A	CCGCAAGCTTCCAGTTATTCTCCTTTAC
3′UTR-RASAL2-S	ACTAACGCGTCGACTTCCAAGGTCAATG
3′UTR-RASAL2-A	CAGAAAGCTTGTCCAACCAGAACCAGTC
3′UTR-TET2-S	CGAGACGCGTAGACTGGTAAAGTGTGGT
3′UTR-TET2-A	CACCGAAGCTTATTCACTATTTCTGCCA
INSIG1-MUT-F	GAAGTTATTAGATGAAAGGTCGCTCTGAATCTTTAAAACAGAC
INSIG1-MUT-R	GTCTGTTTTAAAGATTCAGAGCGACCTTTCATCTAATAACTTC
KCTD15-site1-MUT-F	TGTGGACTCCTCCCAGTTGTCGCTCTATGTGCTTTGCCGGGAG
KCTD15-site1-MUT-R	CTCCCGGCAAAGCACATAGAGCGACAACTGGGAGGAGTCCACA
KCTD15-site2-MUT-F	GAGGGTCCAAAGCTGGCCGTCGCTCCACCAGGGTCCCAGGTGT
KCTD15-site2-MUT-R	ACACCTGGGACCCTGGTGGAGCGACGGCCAGCTTTGGACCCTC
METTL2B-MUT-F	GTCCAGCCTGGGCAAAATTCGCTCTGACCCTGAATCTGAAAGT
METTL2B-MUT-R	ACTTTCAGATTCAGGGTCAGAGCGAATTTTGCCCAGGCTGGAC
NRXN3-MUT-F	AGGAAAAAAACTCAAAACAAATCGCTCTGAGACTATTGCCATA
NRXN3-MUT-R	TATGGCAATAGTCTCAGAGCGATTTGTTTTGAGTTTTTTTCCT
RAB35-MUT-F	CCTCTGAGCGATCAGGCCTCCGGTCGCTCCGTGTGCTTGCAAATTC
RAB35-MUT-R	GAATTTGCAAGCACACGGAGCGACCGGAGGCCTGATCGCTCAGAGGC
RASAL2-MUT-F	AGTAGGAACTGTTGTGTGTCGCTCTCATGAGCCTGTAGGTTCA
RASAL2-MUT-R	TGAACCTACAGGCTCATGAGAGCGACACACAACAGTTCCTACT
TET2-MUT-F	ATGTAGAAGACTCTTATGTCGCTCTTAATGCAGAGAAGGCCTT
TET2-MUT-R	AAGGCCTTCTCTGCATTAAGAGCGACATAAGAGTCTTCTACAT

Underlined characters indicate regions of the miR-720 seed sequences for mutation in luciferase reporter assay

The mutant 3′UTR inserts, with an opposite mutation in the miRNA seed sequence binding sites, were generated by overlapping PCR methods [66] using the mutated primers, listed as Table 1.

Wild-type and mutation inserts were confirmed by DNA sequencing. HEK293T cells were seeded in triplicate at a density of 2 × 10^5^ cells per 24-well plate. The following day, cells were transfected with 100 ng wild-type or mutated firefly reporter vector, 10 ng of the control vector containing Renilla luciferase, and pRL-TK (Promega, US), at a final concentration of 50 nM miRNA, or negative control (NC) precursors, using 1.5 μL Lipofectamine^®^ 2000 (Invitrogen) per well. Cells were assayed 48 h after transfection with the Dual Luciferase Assay (Promega) according to the manufacturer’s instructions. Each experiment was repeated at least in triplicate. The efficiency of transfection was normalized using the Renilla luciferase activity.

### The pcDNA expression plasmids and plasmid transfection

The coding sequence of Rab35 was amplified from HeLa cell cDNA by PCR using primers: forward, 5′-CGCGGATCCATGGCCCGGGACTACGACCA-3′; reverse, 5′-CCGCTCGAGTTAGCAGCAGCGTTTCTTTC-3′. The PCR fragment was inserted using *Bam*H I/*Xho* I into the pcDNA3.0-Vector to generate pcDNA3.0-Rab35. The empty pcDNA3.0-Vector was used as the negative control. HeLa cells were transfected with plasmid using the Lipofectamine^®^ 2000 Transfection Reagent (Invitrogen) according to the manufacturer’s protocol. After 48 h, the cells were harvested for further analysis.

### Western blot analysis

Total cell extracts (40 µg) were resolved on a 10 % SDS-PAGE and transferred to nitrocellulose membranes (Millipore, Germany). Membranes were blocked with 5 % skim milk (BioSharp, US) and further incubated with the following primary antibodies: Rab35 and the mesenchymal cell marker vimentin (1:2000; San Ying Biotechnology, China), the epithelial cell marker E-cadherin (1:200; BD Biosciences, US), and GAPDH (1:10,000; Ray Antibody Beijing, China). After washing, membranes were incubated with a 1:10,000 dilutions of HRP-conjugated secondary antibodies (Santa Cruz Biotechnology, Santa Cruz, CA, USA). After washing six times with TTBS, with each wash lasting 5 min, the membranes were incubated with Pierce ECL western blotting substrate (Thermo Fisher Scientific, US), and the protein bands were visualized by autoradiography and quantified by Image J software (NIH, Bethesda, MD, USA).

### Cell proliferation assay

For the HeLa cell proliferation assay, cells were transiently transfected for 24 h, and then seeded into 96-well plates (6 × 10^3^ cells/well), with each group comprised of six replicates. The cell growth was regarded as the initial value when the cells had been seeded for 2 h. At indicated times, cells were incubated with 10 μL of 5 mg/mL 3-4,5-dimethyl-2-thiazolyl)-2,5-diphenyl-2-H-tetrazolium bromide (MTT) for 4 h at 37 °C, and then the generated crystals were dissolved by dimethyl sulfoxide (DMSO). Absorbance was measured at 450 nm using a microplate reader (Biotech) every 24 h for four consecutive days.

### Wound healing assay

After transfection for 24 h in 6-well plates, cells were replated into 12-well plates (2.5 × 10^5^ cells per well), in triplicate, for the wound healing assay. When the cells reached ~90 % confluency, a 10 µL pipette tip was used to scrap across the confluent cell layer, followed by gentle washing three times with phosphate-buffered saline (PBS), and then addition of culture medium containing 2.5 % fetal bovine serum. The scratch was immediately subjected to photography using a light microscope. After incubation for 24 h, additional photographs were taken. Wound healing ability was determined by measuring the scraped area alteration because of cell migration using Image J software.

### Transwell^®^ assay

After transfection for 24 h, HeLa cells (2.5 × 10^4^ cells) were suspended in 300 µL of Minimal Essential Medium (MEM) without serum and then reseeded into the upper chamber of the Transwell^®^ inserts (8 µm pore size; Corning, USA) of a companion 24-well plate in pre-warmed containing 10 % FBS. After incubating at 37 °C for 24 h, cells on the top surface of the insert were removed by gentle wiping with a cotton swab, and the cells which migrated to the bottom surface of the insert were fixed in 4 % paraformaldehyde for 15 min, stained with 0.1 % crystal violet (Sigma-Aldrich, St. Louis, MO, USA) for 15 min, rinsed in PBS, and then photographed. Cell migration ability was determined by counting the stained cells in the bottom surface of the insert under a light microscope (100×) in eight randomly selected fields. The average number of cells in the eight fields was taken as the final result. Each group had three replicates and each experiment was repeated three times.

### Real-time cell analysis

For real-time cell analysis (RTCA), HeLa cells were transiently transfected for 24 h, and 6 × 10^4^ cells were suspended in 100 µL of MEM without serum in the upper chamber (ACEA Biosciences; Hangzhou, China) according to the basic cell migration assay protocol of the RTCA xCELLigence system (ACEA Biosciences). Each group had four replicates, and the cell migration index was calculated by real-time detection for 48 h.

### Statistical analysis

Data were expressed as mean ± SEM. Student’s *t* test (unpaired, two tailed) was used in the analysis of differences between the experimental groups and control groups, with *p* < 0.05 considered statistically significant. Statistical analyses were performed using GraphPad Prism, version 5.0 software.

## Additional file

10.1186/s13578-015-0047-5 **Table S1. **Results of microarray analysis.
